# Hydrolyzed fish proteins reduced activation of caspase-3 in H_2_O_2_ induced oxidative stressed liver cells isolated from Atlantic salmon (*Salmo salar*)

**DOI:** 10.1186/s40064-015-1432-6

**Published:** 2015-10-31

**Authors:** M. Espe, E. Holen, J. He, F. Provan, L. Chen, K. B. Øysæd, J. Seliussen

**Affiliations:** National Institute of Nutrition and Seafood Research (NIFES), PoBox 2029, 5817 Nordnes, Norway; International Research Institute of Stavanger AS (IRIS), PoBox 8046, 4068 Stavanger, Norway; Fish Nutrition Laboratory, Institute of Aquatic Economic Animals, School of Life Sciences, Sun Yat-Sen University, Guangzhou, China; School of Life Science, East China Normal University, Shanghai, China; Hordafor AS, Salthella, 5397 Bekkjarvik, Norway

**Keywords:** Hydrolyzed proteins, Primary liver cells, Head kidney cells, Co-cultures, Oxidative stress, Viability, Caspase-3, Atlantic salmon

## Abstract

Hydrolyzed fish proteins (H-pro) contains high concentrations of free amino acids and low molecular peptides that potentially benefit health. The following study aimed to test whether the water soluble phase of H-pro could reduce apoptosis and inflammation in primary liver cells isolated from Atlantic salmon following H_2_O_2_ provoked oxidative stress. Cells were grown as monocultures or co-cultured with head kidney cells to assess possible cross talk in inflammation and metabolism during treatments. Cells were grown in media with or without H-pro for 2 days before being stressed with 200 µM H_2_O_2_ then harvested 24 h post exposure. Both treatments were compared to the respective treatments without H_2_O_2_ supplementation. Oxidative stressed cells had increased activation of caspase-3, but supplementation with H-pro in the media prior to the oxidative stress reduced caspase-3 activation. In conclusion, free amino acids and low molecular weight peptides from H-pro attenuated oxidative stress, and made cells able to withstand apoptosis after H_2_O_2_ provoked oxidative stress.

## Background

Cells have to respond quickly to changes in the environment and signal the correct cascade of signals to survive. This cell signaling occurs mainly through acetylation and phosphorylation of peptides or small molecular compounds as Mitogen Activating Phospho Kinases (MAPK’s). MAPK’s are involved in most signal transduction pathways allowing the cells to respond and convert signals rapidly. MAPK signaling pathways are highly conserved through evolution and converts a variety of extracellular stress signals like temperature, irradiation, osmotic shock, cytokines, hormones and inflammation (Brown and Sacks 2009a, b). One of these MAPK is p38MAPK which is known to be strongly activated by cellular stress, in immune response signaling as well as regulation of cell survival (reviewed by Cuadrado and Nebreda 2010). The primary function of p38MAPK activation is the inhibition of cell growth and promotion of both the necrotic and the apoptotic pathways (Kyriakis and Avruch 1996; Wei et al. 2012). It is well known that inflammation and oxidative stress are important factors that regulate pro-inflammatory signaling pathways (reviewed by Pearson et al. 2001). Oxidative stress signals through the Apoptosis Signal-regulating Kinase-1 (ASK-1, a MAPKK) to the p38 MAPK and may increase apoptotic cell death through activation of caspase-3 depending on the time and strength of the signals (Runchel et al. 2011; Czaja 2007). Additionally activation of p38MAPK has been found to release the pro-inflammatory interleukins, TNFα and IL-1β, thus activated p38MAPK concomitantly may increase inflammation (Kyriakis and Avruch 1996).

The water soluble part of hydrolyzed proteins (H-pro) contains free amino acids and several non-protein nitrogen compounds that may have the potential to improve health in fish (Espe and Lied 1999; Liaset et al. 2003; Andersen et al. 2015). One of these being the taurine, which may be low in modern aquaculture diets due to the low inclusion of fishmeal (Espe et al. 2012). We previously showed that supplementation of taurine to primary liver cells isolated from Atlantic salmon improved viability and reduced activation of p-38 MAPK (Espe and Holen 2013). In addition to taurine also several other non-protein compounds like betaine and choline, as well as low molecular weight peptides, are present in hydrolyzed proteins (Samaranayaka and Li-Chan 2011; Klomklao et al. 2013). Betaine supplementation is known to reduce the oxidative stress response (Kwon et al. 2009). Recently, Raspe et al. (2013) reported that free amino acids and dipeptides in combination reduced endotoxemic cytokine production and prevented inflammation better than offered one by one. The current study thus aimed to test whether H-pro, containing several low molecular weight nitrogen compounds and free amino acids could reduce oxidative stress signaling through reduction in the activation of the MAPK cascade resulting in caspase-3 activation. Both liver and head kidney cells were used with the aim to investigate the inflammatory response and oxidative signals following H_2_O_2_ provoked oxidative stress in a co-culture approach as described (Holen et al. 2014).

## Results

### Liver cells

**Fig. 1:**
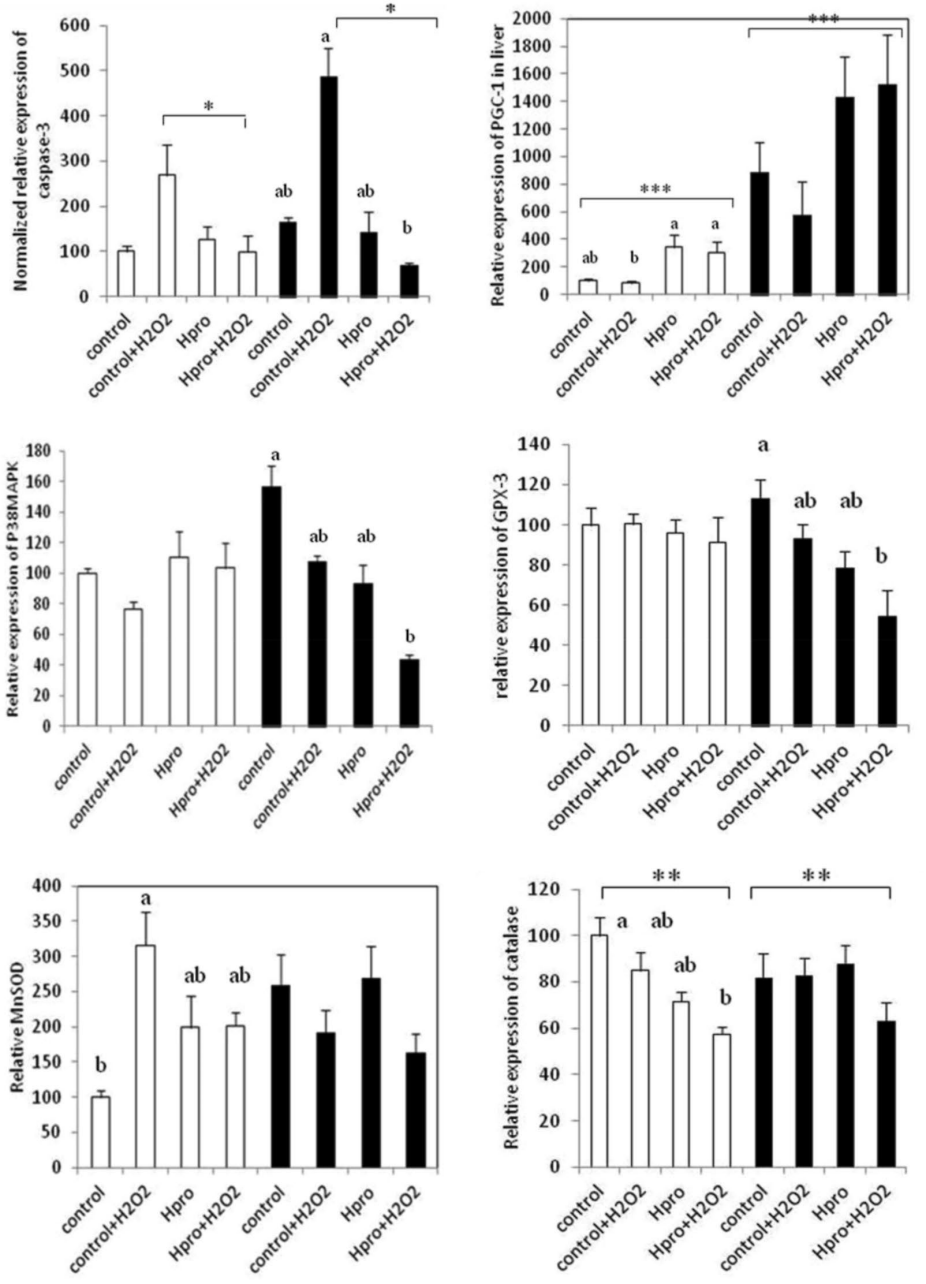
The relative normalized gene expression in liver cells grown as mono and co-cultures. The normalized gene expression of mono-cultured cells grown in the cL-15 media without H_2_O_2_ supplementation (i.e. the positive control) was set equal to 100 and the other treatments calculated relative to these. The *white bars* represent the mono cultured cells, while the *filled bars* represent the co-cultured cells. Differences were assessed by two tailed Mann–Whitney U test and p < 0.05 was accepted as statistically different. Values are mean of 5 fish ± SE. The overall outcome in treatments * conditions (*asterisk*). Also the within each culture condition treatments effects (*letters*) are indicated

**Fig. 2:**
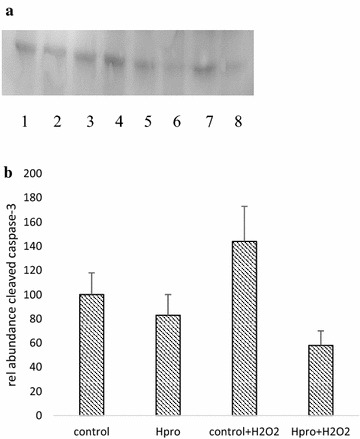
From Western blots it was clear that activated cleaved caspase-3 was more profound in the co-cultured liver cells than in the corresponding mono-cultured liver cells (**a**). *Lanes* *1*–*4* are mono-cultured liver cells while *lanes* *5*–*8* is liver cells co-cultured with head kidney cells. *Lanes 1* and *5* are controls, *lanes 2* and *6* are cells grown in 20 % H-pro, *lanes 3* and *7* are the control cells stressed with 200 µM H_2_O_2_ and *lanes 4* and *8* are the cells grown in 20 % H-pro stressed with 200 µM H_2_O_2._ (**b**) The abundance means of activated cleaved caspase-3 relative to the abundance in the control that are set equal to 100 after the abundance was normalized. Cells grown with H-pro had less active cleaved caspase 3 as compared to controls (p = 0.024, Mann–Whitney U-test n = 5 ± SE)

**Fig. 3:**
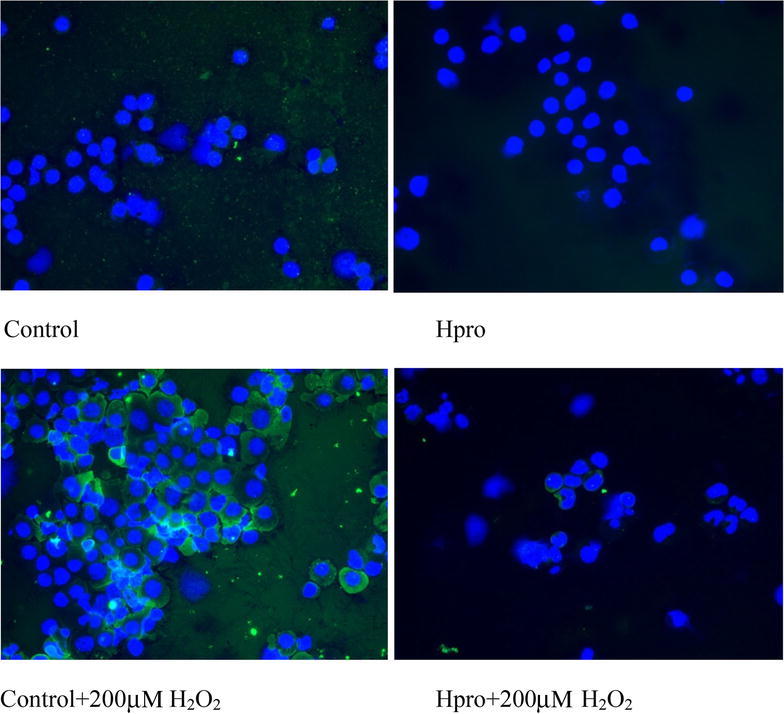
Immunostaining for active cleaved caspase-3 (AbCam, ab77973) verified that cleaved active caspase-3 was higher in the cells cytosol when grown in control media and stressed with 200 µM  H_2_O_2_, while the cells grown in media supplemented with H-pro before being stressed with 200 µM H_2_O_2_ had almost no active cleaved caspase-3. Providing evidence that the free amino acids and low molecular weight peptides of H-pro had the ability to attenuate activation of caspase-3

The relative normalized gene expression of liver cells grown at both culture conditions at the four treatments are shown in Fig. 1. Liver cells grown both as mono- and co-cultured together with head kidney cells showed no difference in gene expression of caspase-3 due to culture conditions (p = 0.13), but cells grown in the cL15 media stressed with H_2_O_2_ had significantly higher gene expression of caspase-3 than the stressed cells grown in media supplemented with H-pro (p = 0.039). The gene expression of p-38MAPK was not affected in either conditions or treatments, but the nuclear PPARγ cofactor 1α (PGC1α) expression was significantly higher in liver cells co-cultured with head kidney cells (p < 0.001). Treatments however, had no impact on PGC1α gene expression (p = 0.39). Of the enzyme analyzed for oxidative protection and detoxification of H_2_O_2_ (GPX-3, MnSOD, catalase) only the gene expression of catalase was affected as stressed cells grown in H-pro media was lower than the unstressed control cells grown in cL-15 (p = 0.01), but variation in between each culture condition did not turn out as statistical different (Fig. 1). The abundance of activated p38MAPK did not differ between treatments (not shown), but activated cleaved caspase-3 was higher in the stressed cells not added H-pro (p = 0.024, Fig. 2b). Of which also was confirmed by the immunohistofluoroscence, which clearly showed that when H-pro was supplemented prior to H_2_O_2_ stress, very little activated caspase-3 was present in the cytosol of the cells (Fig. 3). None of the cytokines (TNFα and NFkB) or the pro-inflammatory interleucines (IL-6, IL-8 and IL-1β) analyzed for gene expression were affected by either treatments or conditions (not shown).

### Head kidney (HK) cells

Independent of cell culture conditions, all HK cells had too low quality and/or concentrations of RNA and was excluded from the analyses of gene expression. When tested for the WB also the bands were too weak to analyze. Thus no analyses could be performed on the HK cultured cells.

## Discussion

It is well known that TNFα induced apoptosis requires activation of the ASK-1-JNK/p38MAPK pathway in mammalian models (reviewed by Matsuzawa and Ichijo 2008) as are oxidative stress (Brieger et al. 2012) and the unfolded protein response (Morishima et al. 2004; Hattori et al. 2009). The fate of the cells however, depends on the strength and length of the activated MAPKs as these are involved in pro as well as anti-survival signaling (i.e. survival and cell differentiation or apoptosis). As little of this is studied in fish, or in cells isolated from fish, the current study aimed to test whether these pathways were involved in sensing the oxidative stress provoked with supplementation of 200 µM H_2_O_2_ in liver and head kidney cells cultured as monocultures and together in co-cultures. The co-culture approach was chosen as we previously experienced that both liver and head kidney cells responded stronger to stress agents when co-cultured as compared to the mono-cultured cells, due to the passage of signaling molecules between the two cultures’ conditions (Holen et al. 2014). Further, these cultures was supplemented with H-pro to assess whether supplementation of H-pro providing several non-protein nitrogen compounds and free amino acids had the capacity to attenuate the oxidative stress and/or modulate the inflammation response in cells and thus may have a beneficial effect on health of animals during periods of increased metabolic stress associated with increased endogenous H_2_O_2_ production (Lieber 1997; Giorgio et al. 2007; Andersen et al. 2013; Muoio and Darrell Neufer 2013). Provided the H-pro has the ability to reduce this signaling cascade they may be beneficial dietary ingredients to be supplemented during periods with increased metabolic stress in animal models (Szarc vel Szic et al. 2010; Ryan and Seeley 2013).

Indeed the H_2_O_2_ supplementation provoked oxidative stress response succeeded and the use of the co-culture approach generally increased the responses obtained as previously reported (Holen et al. 2014), but even though the head kidney cells contained alive cells and proteins, the quality of the RNA was too low to allow any qPCR analysis in either of the culture conditions. Therefore, we only discuss here the mono- and co-cultured liver cells as affected by the provoked oxidative stress to assess that H-pro has the ability to reduce oxidative stress. The increased gene expression of caspase-3 and abundance of active cleaved caspase-3 provoked in H_2_O_2_ stressed cells increased, but when the cells were grown in media containing H-pro liver cells were protected from activation of caspase-3 following the provoked oxidative stress stimuli. Thus, supplementation with H-pro protected the cells from apoptosis by decreasing both the gene expression of caspase-3 (Fig. 1) and activation of active cleaved caspase-3 (Fig. 2). Additionally immunohistochemistry verified that H-pro contained less active cleaved caspase-3 and cells were able to withstand activation of caspase-3 following stress with H_2_O_2_ (Fig. 3).

None of the cytokines (TNFα and NFkB) or the pro-inflammatory interleukines (IL-6, IL-8 and IL-1β) analyzed for gene expression were affected by treatments or conditions in liver cells. As stated above, the head kidney cells contained too little protein and had too bad quality of the RNA to be assessed, we cannot rule out the possibility that these cytokines and pro-inflammatory interleucines are increased in the head kidney cells and involved in the signaling pathway to liver cells in the current model. Any increase in TNFα also signaling through the ASK1, p38 MAPK and caspase-3 cascade in mammalian models (Kyriakis and Avruch 1996; Czaja 2007; Runchel et al. 2011) and may have contributed to the apoptotic response in the model used, especially in the co-cultured liver cells that generally showed higher expressions of genes compared to mono-cultured which was verified by the better response in co-cultures as compared to mono-cultured liver cells. These improvement in responses when co-cultured as compared to mono-cultured cells are in line with our earlier studies on co-cultured liver and head kidney cells stressed with LPS (Holen et al. 2014). PGC1α is a nuclear receptor of which among several other things also led to increased expression of genes to protect cells from oxidative damage and increase energy production in mitochondria (Chen et al. 2011). In addition, the mitochondrial biogenesis improves when PGC1α expression is increased (Onyango et al. 2010). PGC1α over-expression has been reported to directly activate synthesis of anti-oxidative enzymes (Lu et al. 2010). In the current experiment only culture conditions affected the PGC1α expression as the liver cells co-cultured with HK cells had significant higher expression than the mono-cultured cells (p < 0.001) while treatments had no effect. In line with this, the gene expression of GPX-3 or MnSOD were not affected by treatments or conditions. Only the gene expression of catalase was affected as oxidative stressed cells grown in H-pro media was lower than the cells grown in the control media indicative of less H_2_O_2_ needed to be broken down when H-pro providing free amino acids and low weight nitrogen molecules were fed to the cells before the stress insult. This may however not rule out that the enzyme activity of the anti-oxidative enzymes were unaffected, but this warrants new trials.

## Conclusion

It is clear that oxidative stress provoked by H_2_O_2_ supplementation activated caspase-3. Supplementation of free amino acids and low molecular protein fraction of hydrolyzed fish proteins reduced both the transcription and activation of caspase-3 in the liver cells. As such, the water soluble part of H-pro may have the potential to be used to improve health and welfare during stressful periods associated with increased endogenous and exogenous oxidative stress in animal models. Thus, this should be tested using in vivo models.

## Methods

### Preparation of the soluble HPro fraction

The commercial available hydrolyzed fish protein, H-Pro^®^, was used to produce the low molecular weight fish protein hydrolysate (H-pro). H-pro^®^ was solved in distilled water (1:5 v:v) and centrifuged at 50 g for 10 min before the water soluble fraction was collected. The water soluble H-pro fraction was lyophilized and dissolved in its original concentration in Leibovitz’s L-15 media (L-15, Sigma-Aldrich). pH was adjusted to 7.1 before the media was sterile filtrated and used in the cell study as described below.

### Isolation of liver cells

Primary liver cells were isolated from 5 Atlantic salmon with an average BW of 1042 ± 193 g (2 males and 3 females). Isolation of cells was done as described (Espe and Holen 2013). In short liver was perfused with 0.09 M HEPES buffer containing 1.4MNaCl, 0.067 M KCl and 0.03 M EDTA (pH 7.4) until free from blood, before being digested with collagenase (0.1 % dissolved in the HEPES buffer). Cells were squeezed through a Falcon cell strainer and the isolated cells were dissolved in L-15 media, filtered (100 µm filter), before being washed three times with the L-15 media. Cell counts and viability was evaluated using Tryphan Blue (Lonzo Medprobe). Before handling fish were anaesthetized with metocaine (MS222, 5–8 g/L) and killed by a blow to the head. The experimental protocol was approved by the Norwegian Board of Experiments with Living Animals.

### Isolation of head kidney cells

Head kidney cells were isolated from the same 5 fish as the liver cells were isolated from as described (Holen et al. 2014). In short head kidneys were sampled and added a sterile isolation buffer (9 g NaCl/L and 7 g EDTA/L, pH 7.1) before being squeezed through a Falcon cell strainer. The cells were washed twice in L-15 medium and re-suspended in the isolation buffer before being put carefully on top of equal amounts of diluted Percoll (densities 1.08 and 1.06 g/mL). The tubes were spun at 800 g for 20 min, at room temperature, and the cell layer in the interface was collected. Head kidney cells were harvested after centrifugation and solved in the respective media before being counted and assessed for viability as described above.

### The in vitro study

**Fig. 4:**
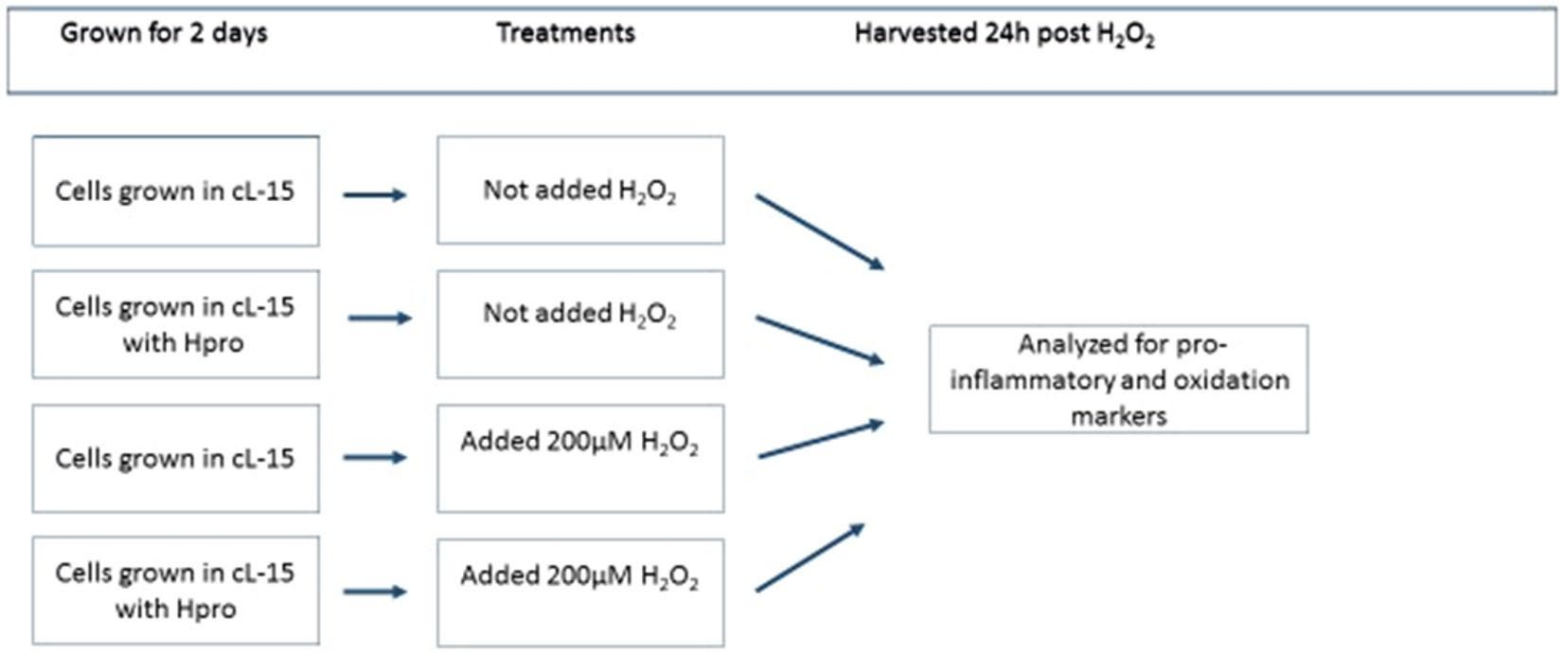
The design chosen for the cell study. The primary liver cells and head kidney cells were grown in complete Leibowitz-15 media (cL-15) that either contained 20 % H-pro (H-pro solved in cL-15 media) or without H-pro supplementation (control). On day two 200 µM H_2_O_2_ was added or not to the cells with the aim to study the ability of H-pro to attenuate H_2_O_2_ induced oxidative stress and inflammation responses in primary liver and head kidney cells cultured as the respective monocultures and together in co-culture

Cells were grown in L-15 medium supplemented with 1 % of 2 mM glutamax 100× (Gibco Life Technologies), 1 % antibiotics (penicillin, 10,000 U/ml, streptomycin, 10,000 µg/ml and amphotericin B, 25 µg/ml, Lonzo Medrobes) and 10 % fetal calf serum (Sigma-Aldrich) to which the H-pro was supplemented or not. When H-pro was added, the soluble fraction of H-pro replaced 20 % of the L-15 medium of the control. Monocultures of liver cells were grown on Laminin coated (1.8 µg/cm^2^, Sigma Aldrich) 6 wells plates (0.8 million cells/cm^2^) grown in darkness in normal atmosphere incubator (Danyo Incubator model MIR-253) for 2 days at 9 ± 1 °C. Mono cultures of head kidney cells were plated in uncoated 6 wells plates in the same manner and concentration as the liver cells. On day 2 both cells grown with L-15 and H-pro media were added 200 µM H_2_O_2_ and compared to the respective controls not added any H_2_O_2_. Cells were harvested 24 h post H_2_O_2_ supplementation. In addition to the mono-cultured cells, liver cells and head kidney cells were plated as co-cultures using inserts for the liver cells placed within the wells containing the head kidney cells to allow communication between the two cell types without being physically in contact with one another as described in details (Holen et al. 2014). The design of the in vitro study is illustrated in Fig. 4.

### Harvesting the cells

Monolayer liver cells without medias were added 300 µl lysis buffer [CellLytic^TM^Mcell lysis reagent (cat no C3228, Sigma Aldrich)]. The lysed cells were centrifuged for 15 min at 10,000*g* and the supernatants were stored at −80 °C until analyzed for protein abundance by Western blotting. Likewise, mono layered liver cells without media were added 600 µl RTL-Plus buffer (RNeasy ^®^Plus kit Qiagen), homogenized using a syringe and frozen at −80 °C until the RNA was extracted. Head kidney cells were treated as the liver cells except the cells were spun at 3000 rpm to separate the cells from the media, before sampled as described above.

### Western blot

Western blots (WB) were run as described (Espe and Holen 2013) with the modifications described in Holen et al. (2014). In short samples were mixed with Laemmli buffer (1:1) and run on precast 10 % SDS-gels using a BioRad Mini Protean^®^ Cell. Thereafter the proteins were blotted on PVDF membranes, blocked and incubated overnight with the antibodies tested (1:1000). The following primary rabbit antibodies were used: cleaved active caspase-3 from AbCam (UK, ab77973), P-p38MAPK (BioNordica Norway, #9215) and β-actin from Cell Signaling (BioNordica, Norway, #4967) for normalization of the blots. Then HRP-linked anti rabbit IgG (Cell Signaling, #7074, 1:500) was used as the secondary antibody. Amersham ECL-Advance™Western Blotting detection kit (GE-Healthcare and Chemi Chemiluminiscence) and Image capture (Syngene, Cambridge) was used to detect the proteins. Protein abundance are given as relative abundance to controls of which were equal to 100.

### Immunofluorescence of active cleaved caspase-3

Mono layered liver cells were cytospun on cover slides (25 g 1 min) after being diluted 1:100 in 10 % PBS containing 1 % BSA. Then cells were treated by 0.1 % TritonX100 in PBS for 15 min to permeate the cell membrane, before being blocked with 0.02 % BSA in PBS for 30 min. This was followed by staining with active caspase-3 (AbCam, cat no #ab77973, 1:50) for 1 h and as a secondary fluorescent antibody goat*rabbit IgG Fluorescin conjugated (AP132F Chemicon, 1:50) giving green fluorescence was used. Cells were washed in 10 % PBS containing 1 % BSA and mounted with a drop of proLong^®^Gold antifade reagent with DAPI (Life Technologies cat no #P36931) before imaged by a spectrophotometer (Olympus U-RFL-T, NIS elements BR4.00.07 Nikon software). The pictures of the blue nucleus and green fluorescent active capase-3 in cytosol were merged. All samples were evaluated at 40X and the same exposure time were used for all images to make them comparable.

### Gene expression analysis

RNA was extracted using RNeasy ^®^Plus kit (Qiagen) according to the manufacturer’s instructions, and frozen at −80 °C for further processing. The quantity and quality of RNA was assessed using the NanoDropSpectrophotometer using the RNA 6000 Nano LabChip^®^ kit (Agilent Technologies, Palo Alto, CA, USA). A two-step real-time quantitative polymerase chain reaction (RT-qPCR) protocol was used to measure the mRNA levels of the target genes in liver cells as described in details elsewhere (Holen et al. 2014; Castro et al. 2013) and Ct values obtained were normalized using the GE Norm tool as described (Olsvik et al. 2005). The probes used are listed in Table 1.

**Table 1:** Primer pairs and gene bank accession number of the gene used for the RT-qPCR assays

Genes	Primer pairs	Accession no
Target genes		
p-38 MAPK		
F	GGC ACA CAG ACG ATG AGA TG	EF123661
R	ACA GCG TTC TGC CAG TGAG	
TNFα		
F	GGC GAG CAT ACC ACT CCT CT	AY848945
R	TCG GAC TCA GCA TCA CCG TA	
IL-1β		
F	GTA TCC CAT CAC CCC ATC AC	NM001123582
R	GCA AGA AGT TGA GCA GGT CC	
Caspase 3		
F	ACAGCA AAG AGC TAG AGG TCC AAC AC	DQ008070
R	AAA GCC AGG AGA CTT TGA CGC AG	
NFkB		
F	CAG CGT CCT ACC AGG CTA AAG AGA T	CA341859
R	GCT GTT CGA TCC ATC CGC ACT AT	
IL-8		
F	GAGCGGTCAGGAGATTTGTC	NM_001140710
R	TTGGCCAGCATCTTCTCAAT	
IL-6		
F	ATG AAGGTT GCT GAG GTA GTG G	NM 001124657
R	TAG CAG TGT TGT CAT GGT TAC TGG	
PGC-1α		
F	GTC AAT ATG GCA ACG AGG CTT C	FJ710605
R	TCG AAT GAA GGC AAT CCG TC	
MnSOD		
F	GTT TCT CTC CAG CCT GCT CTA AG	DY718412
R	CCG CTC TCC TTG TCG AAG C	
Catalase		
F	CCA GAT GTG GGC CGC TAA CAA	Est04a09
R	TCT GGC GCT CCT CCT CATT C	
GPX-3		
F	CCT TCC AGT ACC TGG AGT TGA ATG C	CA345853
R	CTC ATG ATT GTC TCC TGG CTC CTG T	
Reference genes		
ELF1a		
F	TGC CCC TCC AGG ATG TCT AC	AF321836
R	CAC GGC CCA CAGGTA CTG	
ARP		
F	GAA AAT CAT CCA ATT GCT GGA TG	AY255630
R	CTT CCC ACG CAA GGA CAG A	

### Statistical methods

All values are reported as mean ± SE from cells isolated from 5 fish. Any differences between treatments and culture conditions were analyzed by the nonparametric Kruskall Wallis two tailed test (Statistica Stat Soft Inc., version 12). P values less than 0.05 were accepted as statistically different. Within each culture the condition treatments effects were assessed to search for any differences in treatments within the two culture conditions. Western blot analysis were run only on co-cultured liver cells.

## Abbreviations

MAPK: Mitogen Activating Phospho Kinase
ASK-1: Apoptosis Signal-regulating Kinase-1
JNK: Jun N-terminal kinase
GPX: glutathione peroxidase
IL: interleucine
PPARγ: peroxysome proliferator activated receptor γ
PGC1α: PPARγ cofactor 1α
